# Analysis of Rates of Completion, Delays, and Participant Recruitment in Randomized Clinical Trials in Surgery

**DOI:** 10.1001/jamanetworkopen.2022.50996

**Published:** 2023-01-17

**Authors:** Cade Shadbolt, Elise Naufal, Samantha Bunzli, Veronique Price, Siddharth Rele, Chris Schilling, Sharmala Thuraisingam, L. Stefan Lohmander, Zsolt J. Balogh, Philip Clarke, Peter Choong, Michelle Dowsey

**Affiliations:** 1 Department of Surgery, St Vincent’s Hospital Melbourne, The University of Melbourne, Melbourne, Australia; 2 School of Health Sciences and Social Work, Griffith University, Nathan Campus, Queensland, Australia; 3 Physiotherapy Department, Royal Brisbane and Women’s Hospital, Queensland, Australia; 4 College of Health and Medicine, Australian National University, Canberra, Australia; 5 Department of Clinical Sciences, Orthopaedics, Lund University, Lund, Sweden; 6 Department of Traumatology, John Hunter Hospital, University of Newcastle School of Medicine and Public Health, Callaghan, Australia; 7 Health Economics Research Centre, Oxford University, Oxford, England

## Abstract

**Importance:**

Discontinuation and nonpublication are established sources of avoidable waste among surgical trials, but rates of delayed completion and recruiting shortfalls remain unclear.

**Objectives:**

To examine the rate of delayed completion, incomplete enrollment, and discontinuation among randomized clinical trials in surgical populations and the duration of delays and extent of recruiting shortfalls among these trials.

**Design, Setting, and Participants:**

This cross-sectional study examined randomized clinical trials in surgical populations registered on ClinicalTrials.gov between January 1, 2010, and December 31, 2014. Analysis was conducted between October 27, 2021, and June 30, 2022.

**Main Outcomes and Measures:**

The main outcomes were the percentages of trials completed on time or with full enrollment. Delays and recruiting shortfalls were identified by comparing projected enrollment and study timeframes prespecified at the time of registration with the actual study duration and enrollment reported on completion or discontinuation. Absolute and relative differences between planned and actual trial conduct were presented for discontinued trials and those completed with delays or recruiting shortfalls.

**Results:**

In total, 2542 randomized clinical trials in surgical populations were included in the study sample, of which 370 (14.6%; 95% CI, 13.2%-15.9%) were completed both on time and with full enrollment. Approximately 1 in 5 trials (20.4%; 95% CI, 18.9%-22.0%) were completed within their planned timeframe, and 1166 trials (45.9%; 95% CI, 43.9%-47.8%) met their prespecified enrollment target. The median delay among completed trials was 12.2 months (IQR, 5.1-24.3 months) or 66.7% (IQR, 30.1%-135.8%) longer than planned. Among completed trials that did not meet their prespecified enrollment target, the median recruiting shortfall was equivalent to 31.0% (IQR, 12.7%-55.5%) of the planned study sample. A total of 546 trials (21.5%; 95% CI, 19.9%-23.1%) were discontinued. The median time to discontinuation was 26.4 months (IQR, 15.2-45.7 months), and the median recruiting shortfall among discontinued trials was equivalent to 92.7% (IQR, 65.0%-100.0%) of the trial’s prespecified enrollment target.

**Conclusions and Relevance:**

This cross-sectional study found that delayed completion, recruiting shortfalls, and untimely discontinuation were common among surgical trials. These findings highlight the importance of ensuring that investigators and funders do not overestimate the feasibility of planned trials.

## Key Points

**Question:**

What are the rates of delays and incomplete enrollment among randomized clinical trials in surgery?

**Findings:**

In this cross-sectional study of 2542 randomized clinical trials registered on ClinicalTrials.gov between 2010 and 2014, approximately 1 in 5 trials were completed within their planned trial timeframe, with the median delay lasting 12.2 months. Less than half of trials met their prespecified enrollment target upon completion, with the median shortfall accounting for 31.0% of the planned sample size.

**Meaning:**

These findings suggest that greater efforts are needed to ensure that investigators and funders do not overestimate the feasibility of planned trials.

## Teaser

This cross-sectional study explores delayed and incomplete enrollment in surgical randomized clinical trials.

## Introduction

Randomized clinical trials are an indispensable means of improving the quality and safety of care afforded to patients undergoing surgery.^1,2^ However, conducting trials in surgical populations remains challenging, with rates of discontinuation among such trials being 44% to 59% higher than those reported in nonsurgical trials.^3,4^ Among unsuccessful surgical trials, it is well established that practical challenges, such as slow accrual, inadequate trial support, and insufficient funding, are common reasons for discontinuation.^3,4,5^

Trialists may also respond to such challenges by extending study timelines to allow for recruitment targets to be met or by finalizing recruitment before accruing the prespecified number of participants needed to power their analyses. Delays can substantially increase trial costs while reducing the timeliness of results. Moreover, finalizing trials before enrolling the planned sample may result in findings that are of limited clinical value.^6,7^ Prior studies^8,9^ exploring delays and incomplete enrollment among surgical trials have focused exclusively on published studies, masking the disproportionate burden of such inefficiencies among trials that remain unpublished.

As the most comprehensive of the international trial registries, ClinicalTrials.gov provides a valuable resource for studying the efficiency of the surgical trials enterprise. As of 2016, ClinicalTrials.gov accounted for two-thirds of studies on the World Health Organization’s International Clinical Trials Registry Platform.^10^ Currently, ClinicalTrials.gov contains information from approximately 330 000 trials from more than 200 countries.^11^ Rates of registration are assumed to be high because registration is a prerequisite for trial publication in International Committee of Medical Journal Editors member journals and is increasingly mandated by regulators and funders.^10,12^ By leveraging information on surgical trials registered on ClinicalTrials.gov, we aim to present a more complete understanding of (1) rates of delayed completion and incomplete enrollment among randomized clinical trials in surgical populations and (2) the duration of delays and extent of recruiting shortfalls in both completed and discontinued trials.

## Methods

### Study Design and Eligibility

This cross-sectional study included randomized clinical trials involving surgical populations that were registered on ClinicalTrials.gov between January 1, 2010, and December 31, 2014. ClinicalTrials.gov was queried on October 27, 2021, using the keyword *surgery* alongside several indexed synonyms (eTable 1 in Supplement 1). Randomized clinical trials identified by this search were excluded if (1) the trial was registered more than 90 days after the planned start date, (2) the planned completion date was not at least 90 days before our final search, (3) the originally planned duration or enrollment was not reported, (4) the trial was completed without reporting the actual duration of the study or number of participants enrolled, or (5) the trial was listed as having an unknown trial status. Remaining trials were independently evaluated by 2 reviewers (E.N., V.P., or C.S.) to determine whether it examined any form of intervention in a surgical population, defined broadly as patients scheduled to undergo—or who had recently undergone—any procedural intervention that alters the anatomy and requires a skin incision or the use of endoscopic technique. Discrepancies were adjudicated by an additional reviewer (S.R.). We used the Strengthening the Reporting of Observational Studies in Epidemiology (STROBE) checklist for cross-sectional studies when reporting this study.^13^ Ethical review was not required because only publicly available trial-level data were analyzed.

### Data Sources

We used the standard export functionality of the ClinicalTrials.gov and Aggregate Analysis of ClinicalTrials.gov websites to extract information relating to the status, design, and industry sponsorship status of all eligible trials.^14^ Industry sponsorship was identified from the lead sponsor of each study, defined as the “organization or person who initiates the study and who has authority and control over the study,” although trials may receive support from multiple parties.^15^ For completed or discontinued trials, we extracted the actual study start date, completion date, and number of participants enrolled from these sources. Because this in-built export functionality only exports information from the most recent version of each protocol, it provides data on actual rather than planned study duration or enrollment for trials that have concluded.

When amendments have been made to the protocol, originally planned study duration and enrollment data can be manually accessed on ClinicalTrials.gov through the history of changes archive, which provides time-stamped records of any amendments. Because this information cannot be exported in bulk through the current functionality of ClinicalTrials.gov, we wrote a script in R that identified each trial’s original protocol from the history of changes archive and recorded the originally planned start date, completion date, and enrollment target. For a random sample of 5% of studies, 1 reviewer (C.S.) manually compared the information extracted by this script (ie, the output data) to the original protocol as it appears on the ClinicalTrials.gov website (ie, the source data). This manual review detected no discrepancies between the source and output data.

### Trial Status, Duration, and Recruitment Outcomes

We examined discrepancies between planned and actual trial conduct by comparing the most recent version of each trial’s protocol with the plans outlined in their originally specified protocols. Trials were classified as completed on time if the trial status was reported as completed in the registry without exceeding its originally planned duration. Trials were classified as completed with full enrollment if the trial had met its planned enrollment target on being listed as completed on the registry. Trials were considered completed on time with full enrollment if they met both these criteria. Trials were classified as discontinued if the trial was listed as terminated or withdrawn in the most recently posted entry. Finally, because study eligibility criteria required trials to have a planned completion before our final search, eligible trials listed as ongoing were considered ongoing with delays. Less stringent approaches to identifying delays and incomplete enrollment were examined in secondary analyses.

### Statistical Analysis

We summarized the baseline characteristics of included trials by using standard descriptive statistics separately for industry- or non–industry-sponsored trials. We then assessed the percentage of trials in each of the status, duration, and recruitment categories described herein and reported discrepancies between planned and actual trial conduct by industry sponsorship status. Absolute and relative differences between anticipated and actual trial duration are presented as medians (IQRs) for trials completed with delays and discontinued trials. Similarly, differences between anticipated and actual recruitment are presented for discontinued trials and those completed without meeting their recruitment target. To determine whether industry sponsorship was associated with trials being completed on time or with full enrollment, we conducted χ^2^ tests with a 2-sided *P* < .05 used to indicate statistical significance. Data were prepared and analyzed between October 27, 2021, and June 30, 2022, using Stata software, version 16.0 (StataCorp LLC) and R software, version 4.1.2 (R Foundation for Statistical Computing).

## Results

Of 16 940 studies identified by our search, 2542 randomized clinical trials in surgical populations were included in the study sample (eFigure in Supplement 1). Approximately 1 in 6 trials (413 [16.2%]) were industry sponsored, and the number of industry trials registered each year remained relatively stable across the study period (Table 1). Non–industry-sponsored trials were more likely to be excluded from the sample due to having an unknown study status (eTable 2 in Supplement 1). The number of non–industry-sponsored trials registered each year increased from 300 in 2010 to 552 in 2014. More than half of industry-sponsored trials (271 of 413 [65.6%]) were multisite, and only 452 of 2129 (21.2%) of non–industry-sponsored trials recruited from multiples sites. Trials of procedural interventions were more common in non–industry-sponsored trials (498 [23.4%]) than industry-sponsored trials (15 [3.6%]). At the time of initial registration, the median anticipated sample size was 180 (IQR, 75-323) for industry-sponsored trials and 98 (IQR, 53-180) for non–industry-sponsored trials.

**Table 1.** Characteristics of 2542 Randomized Clinical Trials in Surgical Populations Registered on ClinicalTrials.gov Between January 1, 2010, and December 31, 2014^a^

Characteristic	Industry sponsored (n = 413)	Non–industry sponsored (N = 2129)	Total (N = 2542)
Year of registration			
2010	86 (20.8)	300 (14.1)	386 (15.2)
2011	79 (19.1)	373 (17.5)	452 (17.8)
2012	92 (22.3)	445 (20.9)	537 (21.1)
2013	78 (18.9)	459 (21.6)	537 (21.1)
2014	78 (18.9)	552 (25.9)	630 (24.8)
Body system			
Cardiovascular	64 (15.5)	306 (14.4)	370 (14.6)
Digestive	46 (11.1)	411 (19.3)	457 (18.0)
Eye	57 (13.8)	67 (3.1)	124 (4.9)
Female reproductive	17 (4.1)	190 (8.9)	207 (8.1)
Integumentary	51 (12.3)	123 (5.8)	174 (6.8)
Mixed	36 (8.7)	212 (10.0)	248 (9.8)
Musculoskeletal	85 (20.6)	466 (21.9)	551 (21.7)
Other	49 (11.9)	241 (11.3)	290 (11.4)
Respiratory	8 (1.9)	113 (5.3)	121 (4.8)
Intervention type			
Drug	180 (43.6)	749 (35.2)	929 (36.5)
Procedure	15 (3.6)	498 (23.4)	513 (20.2)
Device	88 (21.3)	243 (11.4)	331 (13.0)
Other	35 (8.5)	332 (15.6)	367 (14.4)
Mixed	95 (23.0)	307 (14.4)	402 (15.8)
No. of facilities			
Multisite	271 (65.6)	452 (21.2)	723 (28.4)
Single site	116 (28.1)	1623 (76.2)	1739 (68.4)
Not reported	26 (6.3)	54 (2.5)	80 (3.1)
Masking			
Masked	286 (69.2)	1383 (65.0)	1669 (65.7)
None (open label)	125 (30.3)	745 (35.0)	870 (34.2)
Not reported	2 (0.5)	1 (0.0)	3 (0.1)
Target duration, median (IQR), mo	17.2 (9.2-32.4)	23.3 (12.2-35.5)	22.3 (12.2-35.5)
Target enrollment, median (IQR), No.	180 (75-323)	98 (53-180)	100 (58-200)

^a^ Data are presented as number (percentage) of trials unless otherwise indicated.

### Delayed Completion

Overall, 370 of 2542 trials (14.6%; 95% CI, 13.2%-15.9%) were completed both on time and with full enrollment (Table 2). Characteristics of eligible trials are presented separately according to study status in eTable 3 in Supplement 1. Approximately 1 in 5 trials (20.4%; 95% CI, 18.9%-22.0%) were completed within their planned timeframe, and 23.8% (95% CI, 22.1%-25.5%) required less than 10% longer than planned to reach completion (eTable 4 in Supplement 1). Industry-sponsored trials were no more likely to be completed on time than non–industry-sponsored trials (84 of 413 [20.3%] vs 435 of 2129 [20.4%]; difference, −0.1%; 95% CI, −4.3% to 4.1%; *P* = .97). However, non–industry-sponsored trials also tended to experience longer delays when they were completed outside their target timeframe, with the median delay being 13.2 months (IQR, 5.8-25.3 months) among non–industry-sponsored trials and 9.1 months (IQR, 3.7-19.3 months) among industry-sponsored trials (Table 3). Overall, trials that were completed outside their projected timeframe took a median of 66.7% (IQR, 30.1%-135.8%) longer than planned.

**Table 2.** Status of 2542 Randomized Clinical Trials in Surgery Registered on ClinicalTrials.gov Between January 1, 2010, and December 31, 2014

Trial status^a^	Trials, No. (%)		
	Industry sponsored (n = 413)	Non–industry sponsored (n = 2129)	Total (N = 2542)
Completed on time			
Full enrollment	61 (14.8)	309 (14.5)	370 (14.6)
Partial enrollment	23 (5.6)	126 (5.9)	149 (5.9)
Completed with delays			
Full enrollment	139 (33.7)	657 (30.9)	796 (31.3)
Partial enrollment	77 (18.6)	387 (18.2)	464 (18.3)
Discontinued	87 (21.1)	459 (21.6)	546 (21.5)
Ongoing with delays	26 (6.3)	191 (9.0)	217 (8.5)

^a^ Trial status was determined from data exported on October 27, 2021.

**Table 3.** Comparison of Planned vs Actual Trial Duration Among 1260 Randomized Clinical Trials Completed With Delays

Duration status	Median (IQR)		
	Industry sponsored	Non–industry sponsored	Total
Planned duration, mo	16.3 (10.1-29.4)	18.3 (12.2-28.4)	18.2 (11.2-28.4)
Actual duration, mo	27.4 (16.2-47.2)	35.5 (21.3-53.8)	34.5 (20.4-52.8)
Difference, mo	9.1 (3.7-19.3)	13.2 (5.8-25.3)	12.2 (5.1-24.3)
Difference, %	48.8 (23.4-88.0)	72.0 (31.9-148.2)	66.7 (30.1-135.8)

### Incomplete Enrollment

Among eligible trials, 1166 (45.9%; 95% CI, 43.9%-47.8%) met their prespecified enrollment target, and 1290 trials (50.7%; 95% CI, 48.7%-52.7%) enrolled at least 90% of their planned sample (eTable 5 in Supplement 1). Industry-sponsored trials were not significantly more likely to be completed with full enrollment than non–industry-sponsored trials (200 of 413 [48.4%] vs 966 of 2129 [45.4%]; difference, 3.1%; 95% CI, −2.2% to 8.3%; *P* = .26). Because commercially sponsored trials tended to have larger planned sample sizes, the median recruiting shortfall did not differ between industry- and non–industry-sponsored trials when measured in absolute terms (Table 4). However, non–industry-sponsored trials facing recruiting shortfalls enrolled a smaller proportion of their planned sample size (median shortfall, 32.7%; IQR, 13.6%-56.8%) than industry-sponsored trials (median shortfall, 25.0%; IQR, 8.2%-47.5%).

**Table 4.** Comparison of Planned vs Actual Enrollment Among 613 Randomized Controlled Trials Completed Without Meeting Target Enrollment

Enrollment status	Median (IQR)		
	Industry sponsored	Non–industry sponsored	Total
Planned enrollment, No.	207 (103 to 406)	116 (60 to 210)	120 (68 to 244)
Actual enrollment, No.	124 (53 to 253)	68 (36 to 140)	74 (37 to 159)
Difference, No.	−32 (−114 to −8)	−32 (−84 to −12)	−32 (−90 to −12)
Difference, %	−25.0 (−47.5 to −8.2)	−32.7 (−56.8 to −13.6)	−31.0 (−55.5 to −12.7)

### Trial Discontinuation

By the date of our final query, 546 of 2542 trials (21.5%; 95% CI, 19.9%-23.1%) were discontinued. Among discontinued trials, the median enrollment target was 90 (IQR, 50-195) participants, and the median number of participants enrolled on discontinuation was 7 (IQR, 0-35) (Table 5). The median time to discontinuation was 26.4 months (IQR, 15.2-45.7 months), and the median recruiting shortfall among discontinued trials was equivalent to 92.7% (IQR, 65.0%-100.0%) of the trial’s prespecified enrollment target. Despite obvious recruiting shortfalls, trials were generally discontinued after their planned study completion date.

**Table 5.** Comparison of Planned vs Actual Enrollment and Trial Duration Among 546 Discontinued Trials

Duration and enrollment	Median (IQR)		
	Industry sponsored	Non–industry sponsored	Total
Duration^a^			
Planned, mo	21.3 (9.1 to 34.5)	24.4 (14.2 to 36.5)	24.4 (13.2 to 36.5)
Actual, mo	19.3 (10.1 to 34.5)	28.4 (16.2 to 47.7)	26.4 (15.2 to 45.7)
Difference, mo	0.0 (−10.1 to 9.2)	2.2 (−10.1 to 18.3)	1.7 (−10.1 to 17.2)
Difference, %	0.0 (−48.2 to 70.1)	8.2 (−32.1 to 89.8)	6.8 (−35.0 to 88.7)
Enrollment^b^			
Planned, No.	120 (42 to 225)	88 (50 to 166)	90 (50 to 195)
Actual, No.	23 (5 to 60)	5 (0 to 30)	7 (0 to 35)
Difference, No.	−57 (−175 to −25)	−72 (−140 to −38)	−70 (−150 to −35)
Difference, %	−80.0 (−95.0 to −49.1)	−94.7 (−100.0 to −70.0)	−92.7 (−100.0 to −65.0)

^a^ 121 of 546 discontinued trials were missing data relating to actual trial duration.

^b^ 12 of 546 discontinued trials were missing data relating to actual enrollment.

## Discussion

Our analysis of 2542 studies registered on ClinicalTrials.gov indicates that extensive delays, untimely discontinuation, and incomplete recruitment are commonplace when conducting randomized clinical trials of surgical patients. Although most of the examined trials were completed, fewer than 1 in 6 trials reached their target sample size within their planned timeframe. Moreover, many trials were deemed to be complete despite being underpowered, with almost 1 in 3 completed trials failing to meet their prespecified enrollment targets. Inadequate rates of recruitment were also prominent among discontinued trials, with most of these trials recruiting less than one-tenth of their planned sample despite often delaying termination until after the trial’s projected end date.

Widespread waste associated with trial discontinuation and failures to publish findings from both surgical and nonsurgical trials in a timely fashion has been well documented.^3,4,5,16,17,18^ Policy makers have made concerted efforts to minimize the scientific and economic costs of delayed publication or failure to disseminate study findings, with the implementation of the US Food and Drug Administration Amendments Act 801 final rule in January 2017 mandating the submission of results from many trials to the registry within a year of completing collection for primary outcomes.^19,20^ However, our findings indicate that a pressing need remains for trialists, research funding bodies, and other stakeholders to better understand and respond to broader sources of inefficiency within the clinical trials enterprise.

Inefficiency associated with extensive delays among surgical trials has previously been overlooked. With more than 70% of completed trials examined in this study experiencing delays, often spanning more than a year beyond the planned trial end date, the economic impact of extensive delays to trial timelines is undoubtedly substantial. With the number of randomized clinical trials in surgical populations registered each year increasing by two-thirds during our study period, the overall burden of such delays is likely to increase over time unless substantial progress is made toward ensuring that surgical trials are delivered efficiently.

Methodologic limitations in the design and delivery of surgical trials are well-established sources of research waste,^21,22^ although inadequate enrollment has rarely been investigated in empirical studies of research inefficiency. Inadequately powered trials are at an elevated risk of overlooking important differences among interventions, which may result in costly trials being conducted without meaningfully contributing to advances in medical knowledge.^6,7^ Moreover, adhering to prespecified enrollment numbers and stopping rules is an important bulwark against the discretionary halting of recruitment, which may artificially inflate the significance of findings and limit the credence that can be afforded to the results of these trials even when significant treatment effects are reported.^23,24^

Although our findings indicate that delays and recruiting shortfalls are common among surgical trials, available data did not allow for direct assessment of the reasons for these inefficiencies. Currently, studies that do not meet their recruitment target and those that experience delays are not required to explain these issues on the ClinicalTrials.gov platform.^25^ This contrasts the requirements for trials that are reported as discontinued, which must now lodge a publicly available explanation of the reason for stopping.^25^ Implementing additional data elements on the ClinicalTrials.gov platform to encourage or require trialists to explain any alterations to their original protocol would allow for a thorough investigation of the drivers of inefficiency that have been identified in the current study. Moreover, examining differences in these drivers among industry- and non–industry-sponsored trials may help to explain subtle sponsorship-related differences in trial efficiency.

Our findings support earlier work^3,4,5,26^ that indicates that challenges associated with recruitment are among the most common reason for surgical trials being discontinued. However, the overt waste of research funding by many of these discontinued trials may previously have been underestimated. Contrary to the terms used in earlier studies of trial discontinuation, trials that are terminated or withdrawn are rarely discontinued early.^5^ Rather, these trials recruit a median of only 7.3% of their planned sample over a period of more than 2 years and are generally discontinued slightly after their planned completion date. This finding suggests that in many instances, trial activities are being continued—with additional participants being unnecessarily exposed to the risks of trial participation—well after it is apparent that slow recruitment has rendered a trial infeasible.

Funding bodies have an important role to play in addressing the incentives that promote such inefficient uses of resources by ensuring that the feasibility of a surgical trial is demonstrated before committing considerable material and financial resources to fund full-scale studies.^3^ Explicit requirements for evidence from pilot studies completed before applying for trial funding may help to address these concerns. However, external pilot studies may contribute to downstream inefficiencies associated with reviewing funding for pilot studies and ethical concerns tied to such policies, increasing the number of patients enrolled in studies that are not designed to establish clinically meaningful treatment effects. These concerns may be avoided through a combination of monitoring early trial progress and implementing contingent funding arrangements, which partially fund trials with a commitment to additional funding once feasibility has been established during the early stages of the full-scale study.

Further research into optimal funding strategies should be a priority for trialists, research funding bodies, and other stakeholders that are invested in improving the efficient delivery of surgical trials. Moreover, the findings presented here highlight the need for validated tools to aid in the accurate assessment of trial feasibility before commencing recruitment. To our knowledge, none of the available trial feasibility checklists has been rigorously developed via expert consensus, and none of these tools aims to address the specific challenges faced when conducting surgical trials.^27^ As such, our findings suggest that efforts to rigorously develop and field test a standardized approach to assessing the feasibility of surgical trials—with input from experienced industry and nonindustry experts—presents an opportunity to begin addressing the substantial inefficiencies that currently exist within the surgical trials enterprise.

### Limitations

Several limitations warrant consideration when interpreting our findings. First, identification of studies exclusively from ClinicalTrials.gov may limit the extent to which our findings represent an unbiased reflection of the international clinical trials enterprise.^10^ However, the ClinicalTrials.gov platform is the most comprehensive trial registry available, accounting for more than 60% of trials registered during the study period.^28^ Second, some relevant trials may not have been identified by our search because the query used was constrained by the limited search functionality of the ClinicalTrials.gov platform. Third, we excluded studies with an unknown study status, which indicates that a trial was ongoing when last updated but had not been updated more than 2 years after its planned end date. This may have resulted in the rates of some outcomes being imprecisely ascertained. Fourth, although information submitted to ClinicalTrials.gov undergoes quality control review, to our knowledge, information relating to study duration posted to ClinicalTrials.gov has not previously been the subject of validation studies. Comparisons between registered enrollment data and those reported in published manuscripts have found discrepancies in only 2% to 3% of trials.^29,30^ Finally, the extent to which our findings can be applied to contemporary surgical trials enterprise is limited by our chosen study period (2010-2014). This period was selected to limit the impact of recent changes that were made to data collection on the platform following the US Food and Drug Administration Amendments Act 801 final rule^10,19^ and to ensure that the trials examined were not unduly biased toward studies with shorter durations.

## Conclusions

In this cross-sectional study of randomized clinical trials in surgery, delayed completion, recruiting shortfalls, and untimely discontinuation were common. Few of the examined trials successfully recruited their anticipated sample within their planned timeframe. Although further research is needed to understand what is driving unexpected delays and recruiting shortfalls, these findings highlight the importance of ensuring that investigators and funders do not overestimate the feasibility of planned surgical trials.

## Notes

Supplement 1. **eTable 1.** Detailed Summary of ClinicalTrials.gov Search Results **eFigure.** Flow Diagram Summarizing Identification of Eligible Trials **eTable 2.** Characteristics of 681 Trials Excluded Due to “Unknown” Study Status **eTable 3.** Characteristics of 2542 Eligible Trials, by Trial Status **eTable 4.** Proportions of Trials Completed Within Selected Thresholds for Delays **eTable 5.** Proportions of Trials Completed Within Selected Thresholds for Recruiting Shortfalls

Supplement 2.
Data Sharing Statement
