# Supplementary material for: Analysis of Rates of Completion, Delays, and Participant Recruitment in Randomized Clinical Trials in Surgery
Source: JAMA Netw Open. 2023 Jan 17;6(1):e2250996. doi: 10.1001/jamanetworkopen.2022.50996 (PMC9857498; doi:10.1001/jamanetworkopen.2022.50996)
Supplement: Supplement 1. — eTable 1. Detailed Summary of ClinicalTrials.gov Search Results eFigure. Flow Diagram Summarizing Identification of Eligible Trials eTable 2. Characteristics of 681 Trials Excluded Due to “Unknown” Study Status eTable 3. Characteristics of 2542 Eligible Trials, by Trial Status eTable 4. Proportions of Trials Completed Within Selected Thresholds for Delays eTable 5. Proportions of Trials Completed Within Selected Thresholds for Recruiting Shortfalls [file jamanetwopen-e2250996-s001.pdf]

Supplementary Online Content

Shadbolt C, Naufal E, Bunzli S, et al. Analysis of rates of completion, delays, and participant recruitment in randomized clinical trials in surgery. *JAMA Netw Open*. 2023;6(1):e2250996. doi:10.1001/jamanetworkopen.2022.50996

eTable 1. Detailed Summary of ClinicalTrials.gov Search Results

eFigure. Flow Diagram Summarizing Identification of Eligible Trials

eTable 2. Characteristics of 681 Trials Excluded Due to “Unknown” Study Status

eTable 3. Characteristics of 2542 Eligible Trials, by Trial Status

eTable 4. Proportions of Trials Completed Within Selected Thresholds for Delays

eTable 5. Proportions of Trials Completed Within Selected Thresholds for Recruiting Shortfalls

This supplementary material has been provided by the authors to give readers additional information about their work.

© 2023 Shadbolt C et al. *JAMA Network Open.*

eTable 1. Detailed summary of ClinicalTrials.gov search results

Terms	Search Results*	Entire Database**
Surgery	16,940 studies	70,159 studies
Surgical	7,062 studies	29,615 studies
Operations	2,385 studies	9,778 studies
Surgically	402 studies	1,624 studies
Invasive procedures	183 studies	769 studies
Operative procedures	74 studies	227 studies
Operative therapy	21 studies	61 studies

* Number of studies containing the term or synonym that were first posted to ClinicalTrials.gov between 01/01/2010 and 31/12/2014
** Number of studies in the entire database containing the term or synonym

© 2023 Shadbolt C et al. *JAMA Network Open.*

eFigure. Flow diagram summarizing identification of eligible trials

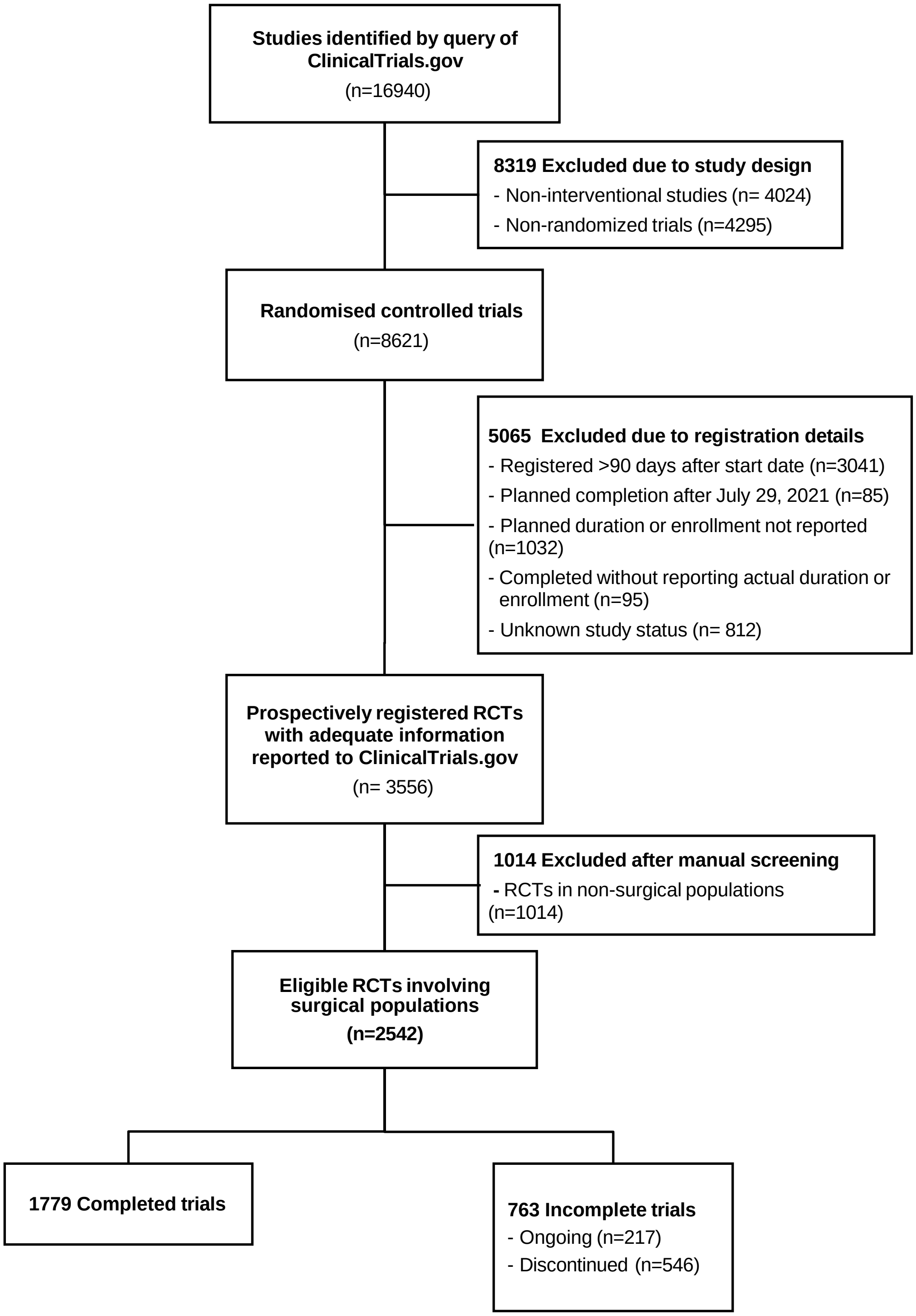

© 2023 Shadbolt C et al. *JAMA Network Open.*

eTable 2. Characteristics of 681 trials excluded due to "Unknown" study status

	Known status	Unknown status*	Total
	N=2,542	N=681	N=3,223
Registration Year			
2010	386 (15.2%)	96 (14.1%)	482 (15.0%)
2011	452 (17.8%)	117 (17.2%)	569 (17.7%)
2012	537 (21.1%)	120 (17.6%)	657 (20.4%)
2013	537 (21.1%)	150 (22.0%)	687 (21.3%)
2014	630 (24.8%)	198 (29.1%)	828 (25.7%)
Body System			
Cardiovascular	370 (14.6%)	90 (13.2%)	460 (14.3%)
Digestive	457 (18.0%)	191 (28.0%)	648 (20.1%)
Eye	124 (4.9%)	32 (4.7%)	156 (4.8%)
Female reproductive	207 (8.1%)	41 (6.0%)	248 (7.7%)
Integumentary	174 (6.8%)	33 (4.8%)	207 (6.4%)
Mixed	248 (9.8%)	53 (7.8%)	301 (9.3%)
Musculoskeletal	551 (21.7%)	122 (17.9%)	673 (20.9%)
Other	290 (11.4%)	79 (11.6%)	369 (11.4%)
Respiratory	121 (4.8%)	40 (5.9%)	161 (5.0%)
Intervention type			
Drug	929 (36.5%)	186 (27.3%)	1,115 (34.6%)
Procedure	513 (20.2%)	211 (31.0%)	724 (22.5%)
Device	331 (13.0%)	75 (11.0%)	406 (12.6%)
Other	367 (14.4%)	97 (14.2%)	464 (14.4%)
Mixed	402 (15.8%)	112 (16.4%)	514 (15.9%)
Lead Sponsor			
Industry	413 (16.2%)	27 (4.0%)	440 (13.7%)
Non-industry	2,129 (83.8%)	654 (96.0%)	2,783 (86.3%)
No. of facilities			
Multi-site	723 (28.4%)	116 (17.0%)	839 (26.0%)
Single site	1,739 (68.4%)	529 (77.7%)	2,268 (70.4%)
Not reported	80 (3.1%)	36 (5.3%)	116 (3.6%)
Masking			
Masked	1,669 (65.7%)	410 (60.2%)	2,079 (64.5%)
None	870 (34.2%)	271 (39.8%)	1,141 (35.4%)
Not reported	3 (0.1%)	0 (0.0%)	3 (0.1%)
Target duration, mo. Med (IQR)	22.3 (12.2-35.5)	24.3 (12.2-36.5)	23.3 (12.2-36.5)
Target enrolment, no. Med (IQR)	100 (58-200)	100 (60-200)	100 (60-200)

* 131/812 studies excluded from study due to reporting an "unknown" study status are not presented here as they would have subsequently been excluded for examining a non-surgical population

© 2023 Shadbolt C et al. *JAMA Network Open.*

eTable 3. Characteristics of 2542 eligible trials, by trial status.

	Completed on time		Completed w/ delays			
	Full enrolment	Partial enrolment	Full enrolment	Partial enrolment	Discontinued	Ongoing w/ delays
	N=370	N=149	N=796	N=464	N=546	N=217
**Registration Year**						
**2010**	54 (14.6)	21 (14.1)	142 (17.8)	70 (15.1)	86 (15.8)	13 (6.0)
**2011**	59 (15.9)	24 (16.1)	134 (16.8)	91 (19.6)	109 (20.0)	35 (16.1)
**2012**	74 (20.0)	40 (26.8)	164 (20.6)	119 (25.6)	101 (18.5)	39 (18.0)
**2013**	73 (19.7)	33 (22.1)	173 (21.7)	93 (20.0)	118 (21.6)	47 (21.7)
**2014**	110 (29.7)	31 (20.8)	183 (23.0)	91 (19.6)	132 (24.2)	83 (38.2)
**Body System**						
**Cardiovascular**	44 (11.9)	18 (12.1)	118 (14.8)	88 (19.0)	67 (12.3)	35 (16.1)
**Digestive**	65 (17.6)	25 (16.8)	133 (16.7)	76 (16.4)	105 (19.2)	53 (24.4)
**Eye**	17 (4.6)	6 (4.0)	44 (5.5)	24 (5.2)	25 (4.6)	8 (3.7)
**Female reproductive**	39 (10.5)	11 (7.4)	79 (9.9)	33 (7.1)	32 (5.9)	13 (6.0)
**Integumentary**	20 (5.4)	14 (9.4)	53 (6.7)	28 (6.0)	43 (7.9)	16 (7.4)
**Mixed**	44 (11.9)	13 (8.7)	73 (9.2)	40 (8.6)	68 (12.5)	10 (4.6)
**Musculoskeletal**	66 (17.8)	30 (20.1)	182 (22.9)	95 (20.5)	130 (23.8)	48 (22.1)
**Other**	51 (13.8)	27 (18.1)	76 (9.5)	49 (10.6)	57 (10.4)	30 (13.8)
**Respiratory**	24 (6.5)	5 (3.4)	38 (4.8)	31 (6.7)	19 (3.5)	4 (1.8)
**Intervention type**						
**Drug**	172 (46.5)	60 (40.3)	301 (37.8)	154 (33.2)	190 (34.8)	52 (24.0)
**Procedure**	70 (18.9)	28 (18.8)	164 (20.6)	79 (17.0)	107 (19.6)	65 (30.0)
**Device**	40 (10.8)	19 (12.8)	110 (13.8)	65 (14.0)	71 (13.0)	26 (12.0)
**Other**	49 (13.2)	25 (16.8)	121 (15.2)	90 (19.4)	68 (12.5)	14 (6.5)
**Mixed**	39 (10.5)	17 (11.4)	100 (12.6)	76 (16.4)	110 (20.1)	60 (27.6)
**Lead Sponsor**						
**Industry**	61 (16.5)	23 (15.4)	139 (17.5)	77 (16.6)	87 (15.9)	26 (12.0)
**Non-industry**	309 (83.5)	126 (84.6)	657 (82.5)	387 (83.4)	459 (84.1)	191 (88.0)
**No. of facilities**						
**Multi-site**	77 (20.8)	36 (24.2)	224 (28.1)	142 (30.6)	141 (25.8)	103 (47.5)
**Single site**	277 (74.9)	111 (74.5)	559 (70.2)	315 (67.9)	363 (66.5)	114 (52.5)
**Not reported**	16 (4.3)	2 (1.3)	13 (1.6)	7 (1.5)	42 (7.7)	0 (0.0)
**Masking**						
**Masked**	281 (75.9)	101 (67.8)	555 (69.7)	300 (64.7)	328 (60.1)	104 (47.9)
**None**	89 (24.1)	47 (31.5)	241 (30.3)	162 (34.9)	218 (39.9)	113 (52.1)
**Not reported**	0 (0.0)	1 (0.7)	0 (0.0)	2 (0.4)	0 (0.0)	0 (0.0)
**Target duration, mo. Med (IQR)**	16.2 (10.1-27.4)	25.4 (13.2-43.6)	15.3 (10.2-25.4)	23.3 (12.2-32.4)	24.4 (13.2-36.5)	48.7 (27.3-70.1)
**Target enrolment, no. Med (IQR)**	82.5 (50-150)	144 (69-286)	90 (50-168)	120 (68-231)	90 (50-196)	168 (78-369)

© 2023 Shadbolt C et al. *JAMA Network Open.*

eTable 4. Proportions of trials completed within selected thresholds for delays

	No. (%) of trials completed below threshold		
**Percentage of planned duration**	**Industry (N=413)**	**Non-industry (N=2129)**	**Total (N=2542)**
**110%**	103 (24.9)	501 (23.5)	604 (23.8)
**125%**	140 (33.9)	640 (30.1)	780 (30.7)
**150%**	193 (46.7)	836 (39.3)	1,029 (40.5)
**175%**	231 (55.9)	970 (45.6)	1,201 (47.2)
**200%**	251 (60.8)	1,071 (50.3)	1,322 (52.0)

eTable 5. Proportions of trials completed within selected thresholds for recruiting shortfalls

	No. (%) of trials completed above threshold		
**Percentage of planned enrollment**	**Industry (N=413)**	**Non-industry (N=2129)**	**Total (N=2542)**
**90%**	229 (55.4)	1,061 (49.8)	1,290 (50.7)
**80%**	243 (58.8)	1,139 (53.5)	1,382 (54.4)
**70%**	254 (61.5)	1,208 (56.7)	1,462 (57.5)
**60%**	271 (65.6)	1,257 (59.0)	1,528 (60.1)
**50%**	275 (66.6)	1,308 (61.4)	1,583 (62.3)

© 2023 Shadbolt C et al. *JAMA Network Open.*
