# Supplementary material for: Analysis of Rates of Completion, Delays, and Participant Recruitment in Randomized Clinical Trials in Surgery
Source: JAMA Netw Open. 2023 Jan 17;6(1):e2250996. doi: 10.1001/jamanetworkopen.2022.50996 (PMC9857498; doi:10.1001/jamanetworkopen.2022.50996)
Supplement: Supplement 2. — Data Sharing Statement [file jamanetwopen-e2250996-s002.pdf]

Data Sharing Statement

Shadbolt. Analysis of Rates of Completion, Delays, and Participant Recruitment in Randomized Clinical Trials in Surgery. *JAMA Netw Open*. Published January 17, 2023. doi:10.1001/jamanetworkopen.2022.50996

Data
Data available: No

Additional Information
Explanation for why data not available: Data from included trials is available through the ClinicTrials.gov and Access to Aggregate Content of ClinicalTrials.gov (AACT) databases
